# Synthesis and Biological Evaluation of New 3-Phenyl-1-[(4-arylpiperazin-1-yl)alkyl]-piperidine-2,6-diones

**DOI:** 10.3797/scipharm.1012-17

**Published:** 2011-02-12

**Authors:** Anna Bielenica, Jerzy Kossakowski, Marta Struga, Izabela Dybała, Roberta Loddo, Cristina Ibba, Paolo La Colla

**Affiliations:** 1 Department of Medical Chemistry, First Faculty of Medicine, The Medical University of Warsaw, 3 Oczki Street, 02-007 Warsaw, Poland; 2 Department of Crystallography, Faculty of Chemistry, Maria Curie-Sklodowska University, 3 Maria Curie-Sklodowska Square, 20-031 Lublin, Poland; 3 Department of Biomedical Science and Technology, University of Cagliari, 09042 Monserrato (CA), Italy

**Keywords:** Antiviral activity, Cytotoxicity, 3-Phenylpiperidine-2,6-dione, X-ray crystallography

## Abstract

A set of 13 alkyl derivatives of 3-phenylpiperidine-2,6-dione were synthesized. Newly obtained compounds were investigated *in vitro* against HIV-1 and other selected viruses. The benzyl **3f** and fluorophenyl **3g** derivatives showed moderate protection against CVB-2 and the compound **3g** also against HSV-1. Derivatives were tested also for their antibacterial and antifungal activity. The molecular structures of **3a** and **3d** were determined by an X-ray analysis.

## Introduction

Literature survey shows that phenylpiperazinyl group is a nuclei of antiviral, antibacterial and antifungal [1–3] agents. It could be integrated with imidazole [4, 5], 1,8-naphthyridone [6], furan [7], 1,4-dithiine [8] or quinolone [9] rings. N-alkyl and N-aryl piperazines are also present as phosphonate [10] and cyano [11] derivatives. Short-chain *p*-substituted aryl piperazines are found as active against *Staphylococcus aureus* and *Pseudomonas aeruginosa* [12, 13]. Recently, derivatives of diarylpiperidin-4-one were found as the new class of antimicrobial agents with activity against pathogenic bacterial species and fungal strains [14]. There is also an increasing concern on substituted 4-arylpiperazines, 4-azatricyclodec-8-ene-3,5-dione derivatives, as some of them have been reported as antiviral agents, e.g. against Yellow Fever Virus (YFV) and Border Disease Virus (CVB-2), as well as antibacterial and antifungal compounds [1, 15]. Some anti-HIV-1 agents, as NNRTIs (non-nucleoside reverse transcriptase inhibitors) possess an arylpiperazine part (Atevirdine, Indinavir, Delavirdine and Vicriviroc). Their activity is also connected with the presence of pyridinylpiperazine or 2-hydroxypropyl fragments. Literature survey revealed that modifications of their structures lead to new potential antiviral drugs [3, 16–19].

This work describes synthesis and a wide spectrum of antiviral activity screening of some novel 3-phenylpiperidine-2,6-diones. Almost all piperazine derivatives presented in this study were tested on anti-HIV-1 activity. Antibacterial and antifungal activity of most of newly synthesized compounds was also evaluated. Due to its high 5-HT_1A_ receptor affinity and selectivity [20], the derivative **3a** was tested for its pharmacological properties in three behavioral tests in mice (data not shown). The molecular structures of **3a** and **3d** were determined by an X-ray analysis.

## Results and discussion

**Fig. 1.**
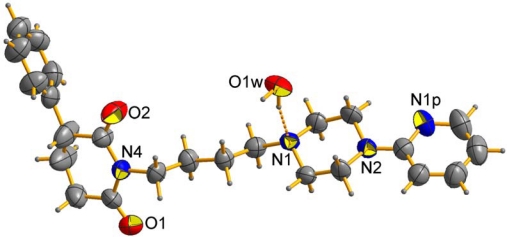
Molecular conformation of **3d** and the O-H…N hydrogen bond (dashed line) fromed between base and water molecule.

**Fig. 2.**
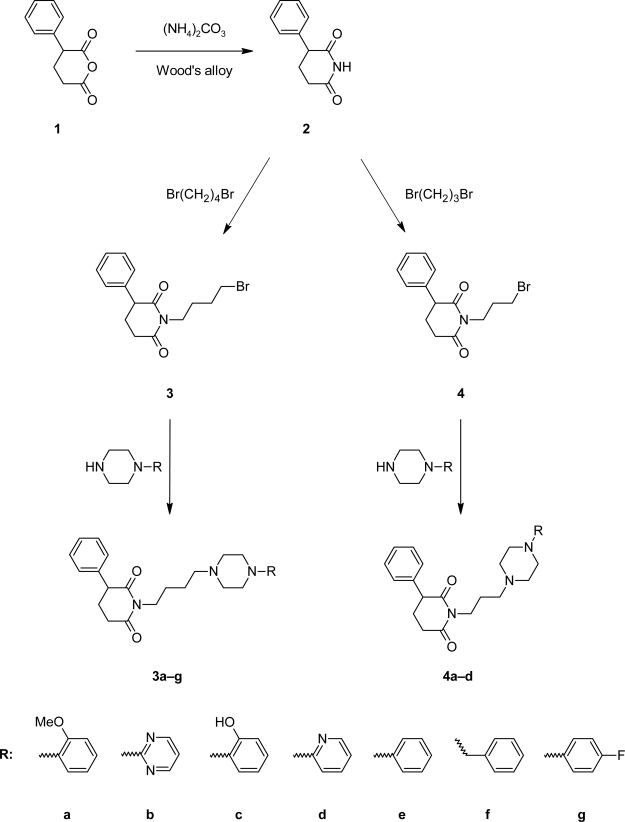
Synthesis of 3-phenylpiperidine-2,6-dione derivatives.

The preparation of new thirteen 3-phenylpiperidine-2,6-dione derivatives is described. The general synthetic pathway and structure of the investigated compounds is given in Fig. 2. The starting imide **2** was obtained from 2-phenylglutaric anhydride in reaction with ammonium carbonate. Next the compound was subjected to the reaction with 1,4-dibromo-butane and 1,3-dibromopropane in order to be transformed into the corresponding alkyl derivatives **3** and **4**. Next these compounds were condensed with appropriate amines to yield compounds **3a–3g** and **4a–4c**. Obtained N-substituted derivatives were purified by column chromatography. Elemental analysis and ^1^H NMR spectra confirmed the identity of the product. The molecular structure of **3a** and **3d** was established by the crystal structure analysis (Fig. 1). Moreover, the two crystals are hemihydrates with a high degree of isostructurality, *viz*. they have the same symmetry, similar mode of molecular packing and noncovalent interactions. Very characteristic feature is: (a) the O–H…N hydrogen bond formed between the water molecule and the piperazine N1-atom, and (b) the lack of hydrophobic interactions of both the methoxy group (**3a**) and pyridine N-atom (**3d**).

For biochemical studies N-substituted alkyl derivatives were converted into their hydrochlorides. Compounds 3a and 3d were presented as monohydrochlorides and **3b**, **3c**, **3e**, **3f**, **3g**, **4a**, **4b**, **4d** as dihydrochlorides.

Ten compounds, N-substituted derivatives of 3-phenylpiperidine-2,6-dione, were evaluated *in vitro* against viruses, bacteria and fungi.

Title compounds were evaluated for antiviral activity against viruses representative of two of the three genera of Flaviviridae family, that is, Flaviviruses (Yellow Fever Virus, YFV) and Pestiviruses (Bovine Viral Diarrhoea Virus, BVDV), as Hepaciviruses can hardly be used in routine cell-based assays. Compounds were also tested against representatives of other virus families. Among ssRNA+ were a retrovirus (human immunodeficiency virus type 1, HIV-1) and two Picornaviruses (Coxsackie Virus type B2, CVB-2 and Poliovirus type-1, Sabin strain, Sb-1); among ssRNA- were a Rhabdoviridae (Vesicular Stomatitis Virus, VSV) representative. Among double-stranded RNA (dsRNA) viruses was a Reoviridae representative (Reo-1). Two representatives of DNA virus families were also included: Herpes Simplex type-1, HSV-1 (Herpesviridae) and Vaccinia Virus, VV (Poxviridae).

In addition to the antiviral activity, compounds were evaluated *in vitro* against representative strains of Gram-positive and Gram-negative bacteria (*Staphylococcus aureus, Pseudomonas aeruginosa*), yeasts and moulds (*Candida albicans* and *Aspergillus niger*).

AZT (3’-azidothymidine), NM 108 (2’-ß-methylguanosine), NM 176 (2’-ethynylcitidine), M 5255 (Mycophenolic Acid) and ACG (acycloGuanosine) were used as reference inhibitors of ssRNA+, ssRNA- and DNA viruses, respectively.

Two of tested derivatives presented moderate antiviral activity (Table 1). The CVB-2 cells were susceptible to benzyl (**3f**) and 1-(4-fluorophenyl)-substituted (**3g**) long-chain arylpiperazine derivatives. The fluorophenyl derivative was also active against HSV-1 virus. The 50% cytotoxic concentration (CC_50_) of this compound in Vero-76 cells was 92 μM. The range of cytotoxicity of compounds in MT-4 cells was from 100 to 54 μM.

Gram-negative rods as well as gram-positive strains and fungal organisms were resistant to all tested agents. Their minimal inhibitory concentration (MIC) values for all compounds were above 100 μM (Table 2). However, none of title compounds turned out to be active against HIV-1, BVDV or representatives of ssRNA- and dsRNA viruses.

Theoretical calculated lipophilicity (log*P*) of synthesized compounds ranged from 1.89 to 3.92. According to Clark and Lobell rules [21], all derivatives could cross the blood-brain barrier to act as ligands of receptors of central nervous system.

## Experimental

### Chemistry

All chemicals and solvents were purchased from Aldrich (Vienna, Austria). Melting points were determined on an Electrothermal Digital Melting Point Apparatus (Essex, UK) and are uncorrected. The ^1^H-NMR spectra were recorded on a Bruker (Rheinstetten, Germany) spectrometer, operating at 400 or 300 MHz. The chemical shift values are expressed in ppm relative to TMS as an internal standard. Elemental analyses were recorded on a CHN model 2400 Perkin-Elmer (Hitachi, Tokyo, Japan). TLC was carried out using silica gel 60 F_254_, layer thickness 0.25 mm (E. Merck, Darmstadt, Germany) and the results were visualized using UV lamp at 254 nm. Column chromatography was carried out using silica gel 60 (200–400 mesh, Merck).

The elemental analyses and ^1^H-NMR spectra, as well as melting points are given for dihydrochlorides (except for compounds **3**, **4**, **3a** and **3d**). Yields are presented for crude products.

Log*P* values for arylpiperazine derivatives were calculated using ChemBioDrawUltra 12.0. (http://www.cambridgesoft.com).

Molecular structure of **3a** and **3d** was confirmed by an X-ray crystallography (Table 3). The intensity data were collected at room temperature with a KM4 diffractometer using graphite monochromated CuKα radiation (λ = 1.54178 Å) and ω – 2θ scan mode. Both compounds crystallized as hemihydrates and their crystals were of poor quality. Structure was solved by the SHELXS-97 program and refined by full-matrix least-squares on *F*^2^ using the SHELXL-97 program [18]. Non-hydrogen atoms were refined with anisotropic displacement parameters, except those of phenyl rings of **3a**. The H-atoms of molecules **3a** and **3d** were positioned geometrically and ‘riding’ model was used in the refinement, while H-atoms of water molecules were located on difference maps. Crystallographic data had been deposited with the Cambridge Crystallographic Data Center.

#### 3-Phenylpiperidine-2,6-dione (**2**)

A mixture of 2-phenylglutaric anhydride (0.0165 mol) and (NH_4_)_2_CO_3_ (0.125 mol) was reduced to powder and heated up to 185 °C using a metal bath of Wood's alloy. When CO_2_ and NH_3_ were completely liberated, 10 cm^3^ of paraffin oil was added and whole mixture was heated until the evolution of gas ceased. The residue was crystallized from hexane and ethyl acetate; mp 143 °C (mp 142–143 from methanol [23]).

### General method for preparation of 1-(alkyl)-3-phenylpiperidine-2,6-diones 3 and 4

A mixture of imide **2** (1.5 g, 0.008 mol), 1,4-dibromobutane (0.02 mol) and 1,3-dibromopropane (0.02 mol), respectively, anhydrous K_2_CO_3_ (1.5 g) and catalytic amount of 98% 1,8-diazabicyclo[5.4.0]undec-7-ene (DBU) were refluxed in acetone for 60–70 h. Then, the solvent was removed on a rotary evaporator and the oily residue was purified by column chromatography (chloroform : methanol 9.5:0.5 vol).

#### 1-(4-Bromobutyl)-3-phenylpiperidine-2,6-dione (**3**)

Yield: 78%, mp 138.5–140 °C. ^1^H NMR (400 MHz, CDCl_3_, TMS): δ 7.32 (m, 5H, ArH), 3.85 (m, 3H), 3.43 (t, *J* = 6.4 Hz, 2H), 2.72 (m, 2H), 2.22 (m, 2H), 1.88 (m, 2H), 1.73 (m, 2H). Anal. Calcd. for C_15_H_18_BrNO_2_: C, 55.57; H, 5.60; N, 4.32. Found: C, 55.47; H, 5.61; N, 4.33.

#### 1-(3-Bromopropyl)-3-phenylpiperidine-2,6-dione (**4**)

Yield: 75%, oil. ^1^H NMR (400 MHz, CDCl_3_, TMS): δ 7.34 (m, 4H), 7.17 (d, *J* = 7.6 Hz, 1H), 3.98 (t, *J* = 6.8 Hz, 2H), 3.84 (dd, *J_1_* = 5.6 Hz, *J_2_* = 8.8 Hz), 3.40 (t, *J* = 6.8 Hz, 2H), 2.73 (m, 2H), 2.26 (m, 2H), 2.17 (m, 2H). Anal. Calcd. for C_14_H_16_BrNO_2_: C, 54.21; H, 5.20; N, 4.52. Found: C, 54.47; H, 5.24; N, 4.55.

### General method for the preparation of 1-[4-aryl/heteroarylpiperazin-1-ylbutyl) (3a–3g) and 1-[3-aryl/heteroarylpiperazin-1-ylpropyl) (4a–4c) derivatives of 3-phenylpiperidine-2,6-dione

A mixture of derivative **3** (0.3 g, 0.001 mol) and **4** (0.3 g, 0.001 mol), respectively, the corresponding amine (0.002 mol), anhydrous K_2_CO_3_ (0.3 g) and catalytic amount of KI was refluxed in acetone for 30 h. Then the mixture was filtered off and the solvent was evaporated. The residue was purified by column chromatography (chloroform : methanol 9.5:0.5 vol) and/or crystallized from hexane.

Obtained compounds were converted into their hydrochlorides. The solid product was dissolved in methanol saturated with gaseous HCl. The hydrochloride was precipitated by addition of diethyl ether. The crude product was crystallized from methanol/ethyl ether.

#### 1-{4-[4-(2-Methoxyphenyl)piperazin-1-yl]butyl}-3-phenylpiperidine-2,6-dione (**3a**)

Mp 210 °C from acetone (mp (salt) from acetone 209–210 °C) [20]. Log*P* = 3.64.

#### 3-Phenyl-1-[4-(4-pyrimidin-2-ylpiperazin-1-yl)butyl]piperidine-2,6-dione (**3b**)

Yield: 70%, mp 210–212 °C. ^1^H NMR (400 MHz, CDCl_3_, TMS): δ 8.30 (d, *J* = 4.8 Hz, 2H), 7.33 (m, 3H), 7.17 (d, *J* = 7.2 Hz, 2H), 6.48 (t, *J* = 4.8 Hz, 1H), 3.85 (m, 7H), 2.72 (m, 2H), 2.52 (m, 4H), 2.43 (m, 2H), 2.22 (m, 2H), 1.60 (m, 4H). Anal. Calcd. for C_23_H_29_N_5_O_2_ · 2HCl · ½ H_2_O: C, 55.34; H, 6.76; N, 14.67. Found: C, 55.07; H, 6.72; N, 14.87. Log*P* = 2.34.

#### 1-{4-[4-(2-Hydroxyphenyl)piperazin-1-yl]butyl}-3-phenylpiperidine-2,6-dione (**3c**)

Yield: 67%, mp 234–236 °C. ^1^H NMR (300 MHz, DMSO, TMS): δ 7.30 (m, 5H), 6.91 (m, 3H), 6.77 (t, *J* = 7.2 Hz, 1H), 5.20 (m, 6H), 4.00 (dd, *J_1_* = 4.8 Hz, *J_2_* = 11.2 Hz, 1H), 3.72 (t, *J* = 6.8 Hz, 2H), 3.14 (m, 4H), 2.82 (m, 1H), 2.65 (m, 1H), 2.23 (m, 1H), 2.06 (m, 1H), 1.73 (m, 2H), 1.73 (t, *J* = 6.8 Hz, 2H). Anal. Calcd. for C_25_H_31_N_3_O_3_ · 2HCl · ½ H_2_O: C, 59.64; H, 6.81; N, 8.35. Found: C, 59.51; H, 6.71; N, 8.31. Log*P* = 3.37.

#### 3-Phenyl-1-[4-(4-pyridin-2-ylpiperazin-1-yl)butyl]piperidine-2,6-dione (**3d**)

Yield: 62%, mp 102–104 °C. ^1^H NMR (400 MHz, CDCl_3_, TMS): δ 8.19 (m, 1H), 7.47 (t, *J* = 7.2 Hz, 1H), 7.33 (m, 4H), 7.17 (d, *J* = 7.2 Hz, 1H), 6.63 (m, 2H, pyridine), 3.84 (m, 3H), 3.59 (m, 4H), 2.75 (m, 2H), 2.60 (m, 4H), 2.46 (m, 2H), 2.23 (m, 2H), 1.61 (m, 4H). Anal. Calcd. for C_24_H_30_N_4_O_2_ · 2HCl · 2H_2_O: C, 55.92; H, 7.04; N, 10.87. Found: C, 56.09; H, 6.74; N, 10.55. Log*P* = 3.14. *Crystal data*: C_24_H_30_N_4_O_2_ · ½ H_2_O, crystal system monoclinic, space group *C*2 with unit cell dimensions: *a* = 18.908(4) Å, *b* = 6.244(1) Å, *c* = 19.087(4)= Å, β = 93.56(3)°, *V* = 2249.1(8)Å^3^, *Z* = 4, D(calcd) = 1.227 g/cm^3^. Independent reflections 4328, final *R* indices [for 1293 reflections with *I* > 2σ (*I*)] *R*1 = 0.038, *wR*2 = 0.0961.

#### 3-Phenyl-1-[4-(4-phenylpiperazin-1-yl)butyl]piperidine-2,6-dione (**3e**)

Yield: 60%, mp 177–179 °C. ^1^H NMR (400 MHz, CDCl_3_, TMS): δ 7.17 (m, 10H, ArH), 3.85 (m, 3H, CH_2_-1’ and H-3), 3.22 (m, 4H, piperidine, H-2,6), 2.74 (m, 2H, CH_2_-4’), 2.63 (m, 4H, piperidine, H-3,5), 2.45 (m, 2H, H-4), 2.20 (m, 2H, H-5), 1.60 (m, 4H, CH_2_-2’ and CH_2_-3’). Anal. Calcd. for C_25_H_31_N_3_O_2_ · 2HCl · H_2_O: C, 60.48; H, 7.11; N, 8.47. Found: C, 60.31; H, 6.79; N, 8.37. Log*P* = 3.76.

#### 1-[4-(4-Benzylpiperazin-1-yl)butyl]-3-phenylpiperidine-2,6-dione (**3f**)

Yield: 55%, mp 248–250 °C. ^1^H NMR (300 MHz, DMSO, TMS): δ 7.64 (m, 2H, Ar, H-3,5), 7.46 (m, 3H, Ar, H-2,4,6), 7.30 (m, 5H, ArH), 4.36 (br.s, 2H, CH_2_-1’), 4.00 (dd, *J_1_* = 4.8 Hz, *J_2_* = 11.2 Hz, 1H, H-3), 3.67 (m, 10H, piperidine, H-2,3,5,6 and H-4), 3.09 (m, 2H, CH_2_-4’), 2.80 (m, 1H, CH_2_-Ar), 2.63 (m, 1H, H-4), 2.19 (m, 1H, H-5), 2.03 (m, 1H, H-5), 1.67 (m, 2H, CH_2_-3’), 1.50 (m, 2H, CH_2_-2’). Anal. Calcd. for C_26_H_33_N_3_O_2_ · 2HCl · ½ H_2_O: C, 62.27; H, 7.24; N, 8.38. Found: C, 62.58; H, 6.92; N, 8.47. Log*P* = 3.42.

#### 1-{4-[4-(4-Fluorophenyl)piperazin-1-yl]butyl}-3-phenylpiperidine-2,6-dione (**3g**)

Yield: 73%, mp 178–180 °C. ^1^H NMR (300 MHz, DMSO, TMS): δ 7.12 (m, 9H), 4.00 (dd, *J_1_* = 4.8 Hz, *J_2_* = 11.2 Hz, 1H), 3.70 (m, 4H), 3.50 (m, 2H), 3.14 (m, 7H), 2.81 (m, 1H), 2.64 (m, 1H), 2.22 (m, 1H), 1.74 (m, 2H), 1.50 (t, *J* = 7.2 Hz, 2H). Anal. Calcd. for C_25_H_30_FN_3_O_2_ · 2HCl · ¾ H_2_O: C, 58.88; H, 6.62; N, 8.24. Found: C, 58.86; H, 6.35; N, 8.16. Log*P* = 3.92.

#### 1-{3-[4-(2-Methoxyphenyl)piperazin-1-yl]propyl}-3-phenylpiperidine-2,6-dione **(4a)**

Yield: 63%, mp 178–180 °C. ^1^H NMR (400 MHz, CDCl_3_, TMS): δ 7.33 (m, 3H), 7.18 (d, *J* = 7.6 Hz, 2H), 7.01 (m, 1H), 6.93 (t, *J* = 6.8 Hz, 2H), 6.86 (m, 1H), 3.93 (t, *J* = 6.8 Hz, 2H), 3.85 (m, 4H), 3.20 (m, 4H), 2.74 (m, 8H), 2.24 (m, 2H), 1.93 (m, 2H). Anal. Calcd. for C_25_H_31_N_3_O_3_ · 2HCl · 2H_2_O: C, 65.60; H, 7.03; N, 7.92. Found: C, 56.87; H, 6.69; N, 8.08. Log*P* = 3.18.

#### 3-Phenyl-1-[3-(4-pyrimidin-2-ylpiperazin-1-yl)propyl]piperidine-2,6-dione (**4b**)

Yield: 60%, mp 214–216 °C. ^1^H NMR (400 MHz, CDCl_3_, TMS): δ 8.32 (d, J = 4.8 Hz, 2H), 7.33 (m, 3H), 7.18 (d, *J* = 7.2 Hz, 2H), 6.52 (t, *J* = 4.0 Hz), 3.88 (m, 7H), 2.76 (m, 8H), 2.23 (m, 2H), 1.96 (m, 2H). Anal. Calcd. for C_22_H_27_N_5_O_2_ · 2HCl · ½ H_2_O: C, 55.58; H, 6.36; N, 14.73. Found: C, 55.43; H, 6.40; N, 14.59. Log*P* = 1.89.

#### 1-{3-[4-(2-Hydroxyphenyl)piperazin-1-yl]propyl}-3-phenylpiperidine-2,6-dione (**4c**)

Yield: 65%, mp 158–160 °C. ^1^H NMR (400 MHz, CDCl_3_, TMS): δ 7.33 (m, 3H), 7.17 (m, 3H), 7.07 (t, *J* = 7.6 Hz, 1H), 6.94 (d, *J* = 7.6 Hz), 6.85 (t, *J* = 7.6 Hz, 1H), 3.93 (t, *J* = 7.2 Hz, 2H), 3.88 (dd, *J_1_* = 5.2 Hz, *J_2_* = 8.8 Hz, 1H), 3.01 (m, 4H), 2.76 (m, 8H), 2.24 (m, 2H), 1.91 (m, 2H). Anal. Calcd. for C_24_H_29_N_3_O_3_ · HCl · 2 ½ H_2_O: C, 58.95; H, 7.21; N, 8.59. Found: C, 59.45; H, 6.84; N, 8.33. Log*P* = 2.92.

#### 3-Phenyl-1-[3-(4-pyridin-2-ylpiperazin-1-yl)propyl]piperidine-2,6-dione (**4d**)

Yield: 70%, mp 116–118 °C. ^1^H NMR (400 MHz, CDCl_3_, TMS): δ 8.19 (d, *J* = 3.2 Hz, 1H), 7.47 (m, 1H), 7.32 (m, 3H), 7.17 (d, *J* = 7.2 Hz, 2H), 6.63 (m, 2H), 3.92 (t, *J* = 7.6 Hz, 2H), 3.84 (dd, *J_1_* = 5.2 Hz, *J_2_* = 9.2 Hz, 1H), 3.56 (m, 4H), 2.72 (m, 2H), 2.58 (m, 4H), 2.47 (t, *J* = 6.8 Hz, 2H), 2.21 (m, 2H), 1.83 (m, 2H). Anal. Calcd. for C_23_H_28_N_4_O_2_ · 2HCl · 2H_2_O: C, 55.09; H, 6.83; N, 11.17. Found: C, 55.48; H, 6.54; N, 10.97. Log*P* = 2.69.

## Microbiological assays

### Compounds

Compounds were dissolved in DMSO at 100 mM and then diluted in culture medium.

### Cells and Viruses

Cell lines were purchased from American Type Culture Collection (ATCC). The absence of mycoplasma contamination was checked periodically by the Hoechst staining method. Cell lines supporting the multiplication of RNA viruses were the following: CD4^+^ human T-cells containing an integrated HTLV-1 genome (MT-4); Madin Darby Bovine Kidney (MDBK); Baby Hamster Kidney (BHK-21) and Monkey kidney (Vero 76) cells.

### Bacterial strains

The antibacterial activity of compounds was tested against collection strains representative of Gram-positive bacteria (*Staphylococcus aureus* DSM 2569) and Gram-negative bacteria (*Pseudomonas aeruginosa* DSM 1117). Antifungal activity was tested against collection strains representative of yeasts (*Candida albicans* DSM 1386) and moulds (*Aspergillus niger* DSM 1988).

### Cytotoxicity Assays

For cytotoxicity tests, run in parallel with antiviral assays, MDBK, BHK and Vero 76 cells were resuspended in 96 multiwell plates at an initial density of 6 × 10^5^, 1 × 10^6^ and 5 × 10^5^ cells/mL, respectively, in maintenance medium, without or with serial dilutions of tested compounds. Cell viability was determined after 48–120 hrs at 37 °C in a humidified CO_2_ (5%) atmosphere by the MTT method. The cell number of Vero 76 monolayers was determined by staining with the crystal violet dye.

For cytotoxicity evaluations, exponentially growing cells derived from human haematological tumors [CD4^+^ human T-cells containing an integrated HTLV-1 genome (MT-4)] were seeded at an initial density of 1 × 10^5^ cells/mL in 96 well plates in RPMI-1640 medium, supplemented with 10% fetal calf serum (FCS), 100 units/mL penicillin G and 100 μg/mL streptomycin. Cell cultures were then incubated at 37 °C in a humidified, 5% CO_2_ atmosphere in the absence or presence of serial dilutions of test compounds. Cell viability was determined after 96 hrs at 37 °C by the 3-(4,5-dimethylthiazol-2-yl)-2,5-diphenyltetrazolium bromide (MTT) method [24].

### Antiviral assay

Activity of compounds against Human Immunodeficiency virus type-1 (HIV-1) was based on inhibition of virus-induced cytopathogenicity in MT-4 cells acutely infected with a multiplicity of infection (m.o.i.) of 0.01. Briefly, 50 μL of RPMI containing 1×10^4^ MT-4 were added to each well of flat-bottom microtitre trays containing 50 μL of RPMI, without or with serial dilutions of test compounds. Then, 20 μL of an HIV-1 suspension containing 100 CCID_50_ were added. After a 4-day incubation, cell viability was determined by the MTT method.

Activity of compounds against Yellow Fever Virus (YFV) and Reo virus type-1 (Reo-1) was based on inhibition of virus-induced cytopathogenicity in acutely infected BHK-21 cells. Activities against Bovine Viral Diarrhoea Virus (BVDV), in infected MDBK cells, were also based on inhibition of virus-induced cytopathogenicity.

BHK and MDBK cells were seeded in 96-well plates at a density of 5 × 10^4^ and 3 × 10^4^ cells/well, respectively, and were allowed to form confluent monolayers by incubating overnight in growth medium at 37 °C in a humidified CO_2_ (5%) atmosphere. Cell monolayers were then infected with 50 μL of a proper virus dilution (in serum-free medium) to give an m.o.i = 0.01. 1 hr later, 50 μL of MEM Earle’s medium, supplemented with inactivated foetal calf serum (FCS), 1% final concentration, without or with serial dilutions of test compounds, were added. After 3–4 days of incubation at 37 °C, cell viability was determined by the MTT method.

Activity of compounds against Coxsackie virus, B-2 strain (CVB-2), Polio virus type-1 (Polio-1), Sabin strain, Vesicular Stomatitis Virus (VSV), Vaccinia Virus (VV) and Herpes Simplex Virus type-1 (HSV-1), in infected Vero 76 cells, was determined by plaque reduction assays in Vero 76 cell monolayers. To this end, Vero 76 cells were seeded in 24-well plates at a density of 2 × 10^5^ cells/well and were allowed to form confluent monolayers by incubating overnight in growth medium at 37 °C in a humidified CO_2_ (5%) atmosphere. Then, monolayers were infected with 250 μL of proper virus dilutions to give 50–100 PFU/well. Following removal of unadsorbed virus, 500 μL of Dulbecco’s modified Eagle’s medium, supplemented with 1% inactivated FCS and 0.75% methyl cellulose, without or with serial dilutions of test compounds, were added. Cultures were incubated at 37 °C for **2** (Sb-1 and VSV) or **3** (CVB-2, VV and HSV-1) and then fixed with PBS containing 50% ethanol and 0.8% crystal violet, washed and air-dried. Plaques were then counted. 50% effective concentrations (EC_50_) were calculated by linear regression technique.

### Antibacterial and antifungal assays

The antibacterial and antifungal activities were evaluated by determining the Minimum inhibitory concentration (MIC) by the broth microdilution procedure.

Bacterial strains were grown on Tryptic soy agar at 37 °C for 1 day. Cell suspensions of these recent cultures were prepared in sterile 0.85% saline solution by 4–5 colonies. The turbidity of the suspensions was adjusted to the McFarland 0.5 standard. Suspensions were diluted in cation-supplemented Mueller-Hinton broth. For each microorganism, 100 μL of the fivefold serial dilutions of the compounds in cation-supplemented Mueller-Hinton broth and 100 μL of *inoculum* were added to each well of a microdilution plate (final titre 5 × 10^5^ CFU/mL). The inoculated plates were incubated at 37 °C in non-CO_2_ incubator and humid atmosphere. The MICs were determined after 16–20 h [25].

Fungal strains were grown on Sabouraud’s dextrose agar at 35 °C for 1–5 days. Suspensions of these recent cultures were prepared in sterile saline solution (NaCl 0.85%). Suspensions were then diluted in Sabouraud’s dextrose broth. 100 μL of the fivefold serial dilutions of the compounds in Sabouraud’s dextrose broth and 100 μL of *inoculum* were added to each well of a microdilution plate (*C. albicans* 1 × 10^4^ cell/mL; *A. niger* OD_600_ 0.05). The inoculated plates were incubated at 35 °C in non-CO_2_ incubator and humid atmosphere. The MICs were determined after 24 and 48 h.

The concentration of each *inoculum* was confirmed by viable counts on agar plates by plating the appropriate dilution of the growth control well, immediately after inoculation, and incubating until visible growth. MIC corresponded to the lowest concentration of an antimicrobial compound that showed complete growth inhibition.

### Linear regression analysis

Viral and cell growth at each drug concentration was expressed as percentage of untreated controls and the concentrations resulting in 50% (EC_50_, CC_50_) growth inhibition were determined by linear regression analysis.

## Figures and Tables

**Tab. 1.** Cytotoxicity and antiviral activity of compounds **2**, **3a–3g** and **4a–4d**.

#	**^a^MT-4** CC_50_	**^b^HIV-1** EC_50_	**^c^MDBK** CC_50_	**^d^BVDV** EC_50_	**^e^BHK-21** CC_50_	**^f^YFV** EC_50_	**^f^Reo-1** EC_50_

2	>100	>100	>100	>100	>100	>100	>100
3a	>100	>100	>100	>100	>100	>100	>100
3c	>100	>100	>100	>100	>100	>100	>100
3d	>100	>100	>100	>100	>100	>100	>100
3e	74	>74	>100	>100	>100	>100	>100
3f	>100	>100	>100	>100	>100	>100	>100
3g	54	>54	>100	>100	>100	>100	>100
4a	>100	>100	>100	>100	>100	>100	>100
4b	>100	>100	>100	>100	>100	>100	>100
4c	>100	>100	>100	>100	>100	>100	>100
4d	>100	>100	>100	>100	>100	>100	>100
AZT^§^	50	0.01					
NM 108^#^				1.8		2.5	

#	**^g^Vero-76** CC_50_	**^h^HSV-1** EC_50_	**^h^VV** EC_50_	**^h^VSV** EC_50_	**^h^CVB-2** EC_50_	**^h^Sb-1** EC_50_	
	
2	98	>98	>98	>98	>98	>98	
3a	>100	>100	>100	>100	>100	>100	
3c	>100	>100	>100	>100	>100	>100	
3d	98	>98	>98	>98	>98	>98	
3e	>100	>100	>100	>100	>100	>100	
3f	96	>96	>96	>96	44	>96	
3g	92	31	>92	>92	44	>92	
4a	>100	>100	>100	>100	>100	>100	
4b	98	>98	>98	>98	>98	>98	
4c	>100	>100	>100	>100	>100	>100	
4d	>100	>100	>100	>100	>100	>100	
NM 176^*^					23	18	
M 5255^**^			1.8				
ACG^***^		3					

Antiviral activity is given as EC_50_ (Median Effective Concentration – the concentration of a drug (μM) required to induce a 50% effect), and cytotoxicity is given as CC_50_ (Cytotoxic Concentration – the amount of a drug (μM) at which 50% of cells become dead).

a,c,e,g Compd. concn. (μM) required to reduce the viability of mock-infected MT-4 (CD4^+^ Human T-cells containing an integrated HTLV-1 genome, ^a^) cells or MDBK (Bovine normal kidney, ^c^) cells or BHK (Hamster normal kidney fibroblast, ^e^) monolayers or VERO-76 (Monkey normal kidney) monolayers by 50%, as determined by the colorimetric MTT method.

b,d,f,h Compd. concentration (μM) required to achieve 50% protection of MT-4 cells from the HIV-1-induced cytopathogenicity (^b^) or MDBK cells from the BVDV (Bovine Viral Diarrhea Virus)-induced cytopathogenicity (^d^) or BHK (Kidney fibroblast) cells from the YFV (Yellow Fever Virus) and Reo (Reovirus 1)-induced cytopathogenicity (^f^) or to reduce the plaque number of HSV-1 (Herpesvirus 1), VV (Vaccinia Virus), VSV (Vesicular Stomatitis Virus), CVB-2 (Coxsackievirus B2), Sb-1 (Poliovirus 1) and RSV (Respiratory Syncytial Virus) by 50% in VERO-76 monolayers (^h^), as determined by the MTT method.

§ 3’-azidothymidine;

# 2’-ß-methylguanosine;

* 2’-ethynyl-D-cytidine;

** mycophenolic acid;

*** acycloGuanosine.

**Tab. 2.** Antibacterial and antifungal activities of 3-phenylpiperidine-2,6-dione and its derivatives.

**MIC^a^** **[μM]**				
	***S. aureus*** **DSM 2569**	***P. aeruginosa*** **DSM 1117**	***C. albicans*** **DSM 1386**	***A. niger*** **DSM 1988**
**2**, **3a**, **3c–3g**, **4a–4d**	>100	>100	>100	>100
Ciprofloxacin^b^	4	0.8	–	–
Miconazole^b^	–	–	0.8	20

a The antimicrobial activity is given as Minimum inhibitory concentration (MIC) corresponding to the lowest concentration of an antimicrobial compound that showed complete growth inhibition.

b Ciprofloxacin was solubilized in water (0.1 M solution) and Miconazole in DMSO (0.1 M solution), according to the British Society for Antimicrobial Chemotherapy (BSAC) protocol and stored at 4°C overnight. Reference Compounds were diluted from 100 to 0.0013 μM.

**Tab. 3.** Crystal data and parameters of the data collection and refinement for crystals of **3a**·½·H_2_O and **3d**·½H_2_O.

**Identification code**	**3a**·½ **H_2_O**	**3d**·½ **H_2_O**
No CCDC*	770 127	770 128
Formula weight	444.56	415.53
Crystal system	monoclinic	monoclinic
Space group	*C*2	*C*2
Unit cell dimensions		
*a* (Å)	26.891(5)	18.908(4)
*b* (Å)	6.344(1)	6.244(1)
*c* (Å)	18.631(4)	19.087(4)
β (°)	129.05(3)	93.56(3)
Volume (Å^3^); Z	2468.3(8); 4	2249.1(8); 4
Density (calc) (g cm^−3^)	1.196	1.227
Absorption coeff. (mm^−1^)	0.639	0.646
F(000)	956	892
Crystal size (mm)	0.28 × 0.12 × 0.05	0.22 × 0.20 × 0.04
Theta range for data collection (°)	3.05 to 70.16	4.69 to 72.13
Index ranges	−32 ≤ *h* ≤ 32, −7 ≤ *k* ≤ 7, −16 ≤ *l* ≤ 22	−23 ≤ *h* ≤ 23, −7 ≤ *k* ≤ 7, −23 ≤ *l* ≤ 0
Reflections collected	4852	4462
Independent reflections	4436 [*R*(int) = 0.1722]	4328 [*R*(int) = 0.0489]
Data / restraints / parameters	4436 / 1 / 223	4328 / 1 / 276
Goodness-of-fit on *F*^2^	1.000	0.942
Final *R* indices [*I* >2s (*I*)]	*R*1 = 0.0787,	*R*1 = 0.0380,
	*wR*2 = 0.2201	*wR*2 = 0.0961
Δρ max.; min. (e Å^−3^)	0.25; −0.29	0.18; −0.18

These data can be obtained free of charge from The Cambridge Crystallographic Data Centre via www.ccdc.cam.ac.uk.
